# Enhanced transport of plant‐produced rabies single‐chain antibody‐RVG peptide fusion protein across an *in cellulo* blood–brain barrier device

**DOI:** 10.1111/pbi.12719

**Published:** 2017-04-19

**Authors:** Waranyoo Phoolcharoen, Christophe Prehaud, Craig J. van Dolleweerd, Leonard Both, Anaelle da Costa, Monique Lafon, Julian K‐C. Ma

**Affiliations:** ^1^ Institute for Infection and Immunity St. George's Hospital Medical School University of London London UK; ^2^ Pharmacognosy and Pharmaceutical Botany Faculty of Pharmaceutical Sciences Chulalongkorn University Bangkok Thailand; ^3^ Unité de Neuroimmunologie Virale Département de Virologie Institut Pasteur Paris France

**Keywords:** rabies virus, single‐chain antibody, blood–brain barrier, antibody engineering, plant biotechnology

## Abstract

The biomedical applications of antibody engineering are developing rapidly and have been expanded to plant expression platforms. In this study, we have generated a novel antibody molecule *in planta* for targeted delivery across the blood–brain barrier (BBB). Rabies virus (RABV) is a neurotropic virus for which there is no effective treatment after entry into the central nervous system. This study investigated the use of a RABV glycoprotein peptide sequence to assist delivery of a rabies neutralizing single‐chain antibody (ScFv) across an *in cellulo* model of human BBB. The 29 amino acid rabies virus peptide (RVG) recognizes the nicotinic acetylcholine receptor (nAchR) at neuromuscular junctions and the BBB. ScFv and ScFv‐RVG fusion proteins were produced in *Nicotiana benthamiana* by transient expression. Both molecules were successfully expressed and purified, but the ScFv expression level was significantly higher than that of ScFv‐RVG fusion. Both ScFv and ScFv‐RVG fusion molecules had potent neutralization activity against RABV *in cellulo*. The ScFv‐RVG fusion demonstrated increased binding to nAchR and entry into neuronal cells, compared to ScFv alone. Additionally, a human brain endothelial cell line BBB model was used to demonstrate that plant‐produced ScFv‐RVG^P^ fusion could translocate across the cells. This study indicates that the plant‐produced ScFv‐RVG^P^ fusion protein was able to cross the *in cellulo* BBB and neutralize RABV.

## Introduction

Rabies remains a major burden in resource‐limited countries particularly in Asia and Africa, accounting for approximately 60 000 deaths per year, mainly in children (Fooks *et al*., 2014). The most common source of infection is from an animal bite. After a period of replication in muscle, the virus gains access to the peripheral nervous system before entering the central nervous system (CNS) (Hemachudha *et al*., 2002) by a process of retrograde axonal transport. The virus spreads rapidly to the brain, resulting in an overwhelming encephalitis that kills the host (Hemachudha *et al*., 2002; Lewis *et al*., 2000). Rabies is unique in that once a productive infection has been established in the CNS, the outcome is invariably fatal.

Rabies postexposure prophylaxis (PEP) is highly effective if correctly administered promptly after a potential exposure (Shantavasinkul and Wilde, 2011; Uwanyiligira *et al*., 2012). However, in the case of delayed treatment and the onset of symptoms, PEP is ineffective. RABV antibodies are unlikely to offer therapeutic benefits once RABV has entered the CNS as they cannot cross the blood–brain barrier (BBB) (Pardridge, 2010).

Nicotinic acetylcholine receptors (nAchRs) are ligand‐gated channels located in the neuromuscular junction and in the CNS (Lentz *et al*., 1988). nAchRs facilitate RABV entry into both muscle and neuronal cells (Burrage *et al*., 1985; Lentz *et al*., 1982). The rabies glycoprotein, which forms spikes on the surface of the virus, contains a short motif which interacts with nAchR to mediate entry into cells (Lentz, 1990; Lentz *et al*., 1987). Previous studies have shown that a linear 29 amino acid peptide derived from the rabies glycoprotein (RVG) binds to the alpha subunit of nAchR enabling the delivery of conjugated molecules into the CNS, including siRNA (Kumar *et al*., 2007), nanoparticles (Hwang do *et al*., 2011; Kim *et al*., 2013) and enzymes (Fu *et al*., 2012; Xiang *et al*., 2011).

The objective of this study was to engineer a RABV‐specific antibody that was capable of crossing the BBB to neutralize RABV infection in the CNS. Monoclonal antibody (mAb) 62‐71‐3 IgG is a potent rabies neutralizing antibody (Muller *et al*., 2009). Recombinant IgG and single‐chain antibody (ScFv) of 62‐71‐3 was recently expressed in plants and potent RABV neutralization was demonstrated (Both *et al*., 2013). The ScFv was developed further here to link the RVG peptide using a gene encoding 62‐71‐3. ScFv genetically fused with RVG was cloned, expressed in *Nicotiana benthamiana* and purified by Ni‐affinity chromatography. This molecule was investigated for RABV neutralization and binding to nAchR. The results demonstrate that the RVG peptide does not affect RABV neutralization, but does facilitate nAchR binding and transport of the rabies ScFv across an *in cellulo* BBB model.

## Results

### Expression of 62‐71‐3 ScFv and ScFv ‐RVG fusion

**Figure 1:**
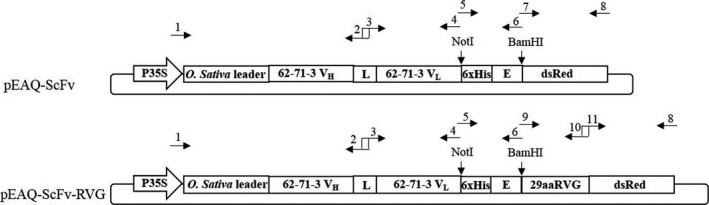
Schematic representation of the T‐DNA regions of the vectors used in this study (Both *et al*., 2013). P35S: CaMV 35S promoter, *Oryza sativa* leader: *O. sativa* leader sequence, 62‐71‐3 V_H_ : variable region of the heavy chain of 62‐71‐3 monoclonal antibody, L: the (Gly_4_Ser)_3_ linker, 62‐71‐3 V_L_ : variable region of the light chain of 62‐71‐3 monoclonal antibody, dsRed: red fluorescent protein from Discosoma sp., 29aaRVG: the 29 amino acid peptide (RVG) from RABV glycoprotein, 6xHis: 6 histidine residues, E: GAPVPYPDPLEPR peptide sequence, the sequences of primers number 1–11 were listed in Table S1.

**Figure 2:**
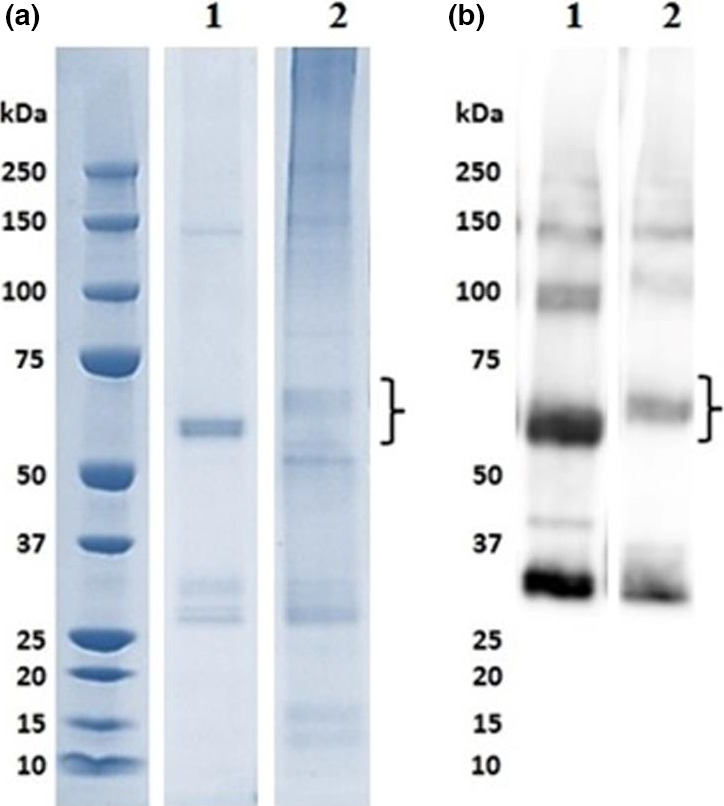
SDS‐PAGE and Western blot analyses of ScFv and ScFv‐RVG fusion proteins. The plant‐produced ScFv^P^ (lane 1) and ScFv‐RVG^P^ fusion proteins (lane 2) were purified by Ni‐affinity chromatography. ScFv and ScFv‐RVG fusion proteins were analysed by SDS‐PAGE under reducing conditions, followed by (a) staining with Coomassie blue or (b) blotting onto nitrocellulose and probing with a mouse anti‐E tag antiserum. The expected size of the ScFv and ScFv‐RVG fusion is approximately 56 kDa and 61 kDa, respectively, which are indicated by curly braces.

ScFv and ScFv‐RVG fusion genes were cloned into the pEAQ vector (Peyret and Lomonossoff, 2013) as shown in Figure 1, and the proteins were expressed in *N*. *benthamiana*. A time‐course of the protein expression between days 4–7 postinfiltration indicated day 6 was the optimal day to harvest (data not shown). The expression level of ScFv and ScFv‐RVG was approximately 100 and 2 μg/g fresh leaf weight, respectively. The Ni‐affinity purified ScFv fusion and ScFv‐RVG fusion were assessed by Coomassie‐stained SDS‐PAGE gel (Figure 2a) or by immunoblotting with anti‐E tag antiserum (Figure 2b). The amounts of purified proteins were quantified by comparing the band intensity with standard BSA protein (MW 66 kDa). Major bands were observed at the expected sizes for ScFv and ScFv‐RVG fusion of 56 kDa (lane 1) and 61 kDa (lane 2), respectively. The identity of the bands was confirmed by Western blot (Figure 2b), which also demonstrated the presence of higher molecular weight bands (probably aggregates) and lower molecular weight bands (possibly degradation products). Of note, the ratio of full‐length protein over degraded protein as shown in the immunoblotting (Figure 2b) is similar for ScFv and ScFv‐RVG.

### Neutralization of rabies virus

**Figure 3:**
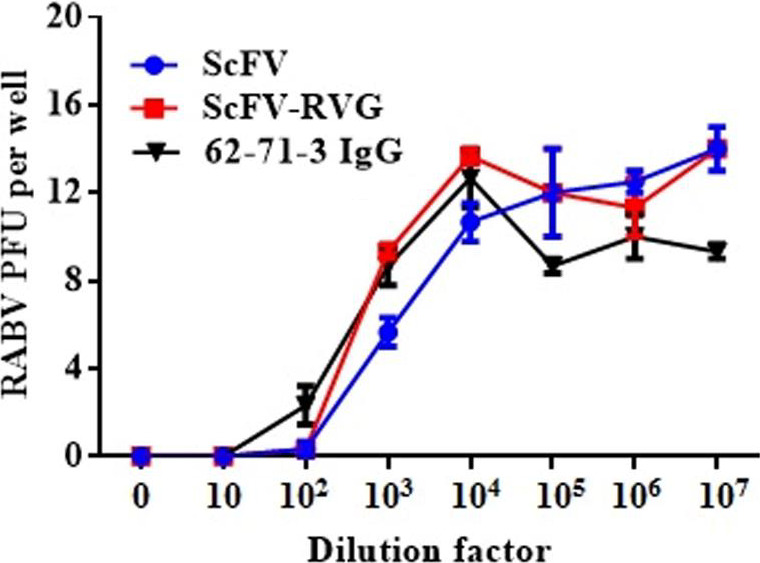
RABV neutralization of ScFv and ScFv‐RVG fusion compared to 61‐71‐3 IgG. The neutralization assay was performed by the rapid fluorescent focus inhibition test on BSR cells. The starting concentration of antibodies was 0.5 mg/mL. Data presented are average values from three independent experiments, and the error bars indicate the standard deviation (SD). Statistical significance was determined by one‐way ANOVA (GraphPad Prism, version 7.0).

The two versions of 62‐71‐3 ScFv were tested to determine their ability to neutralize RABV (ERA strain) *in cellulo* using a plaque‐inhibition assay. With a starting concentration of 0.5 mg/mL, the neutralizing activity of ScFv and ScFv‐RVG fusion was identical to the neutralizing activity of 62‐71‐3 IgG (Figure 3). Statistical analysis by one‐way ANOVA (GraphPad Prism, GraphPad Software, Inc. La Jolla, California, USA, version 7.0) confirmed that there was no significant difference among 62‐71‐3 IgG, ScFv and ScFv‐RVG neutralizing activities.

### Binding to nAchR

**Figure 4:**
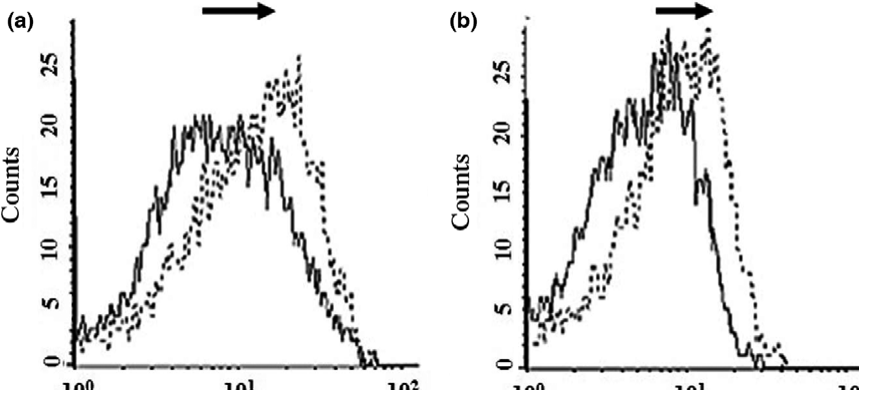
Binding and penetration of 62‐71‐3 ScFv to 293 cells overexpressing nAchR by flow cytometry. Binding (a) and entry (b) were detected with mouse anti‐E antiserum and cy5‐conjugated goat anti‐mouse IgG antiserum. Solid line: ScFv, dotted line: ScFv‐RVG fusion protein. The arrows represent the shift to the right of ScFv‐RVG (dotted line) compared to ScFv (solid line).

Binding and penetration of ScFv and ScFv‐RVG fusion of 293 cells overexpressing nAchR were tested by flow cytometry. A greater proportion of ScFv‐RVG fusion (dotted line) bound to the 293 cells as evidenced by the shift to the right of the dotted line compared to ScFv (solid line), shown in Figure 4a. A greater amount of total ScFv‐RVG fusion (dotted line) was also found in the 293 cells overexpressing nAchR compared to ScFv (solid line, Figure 4b).

**Figure 5:**
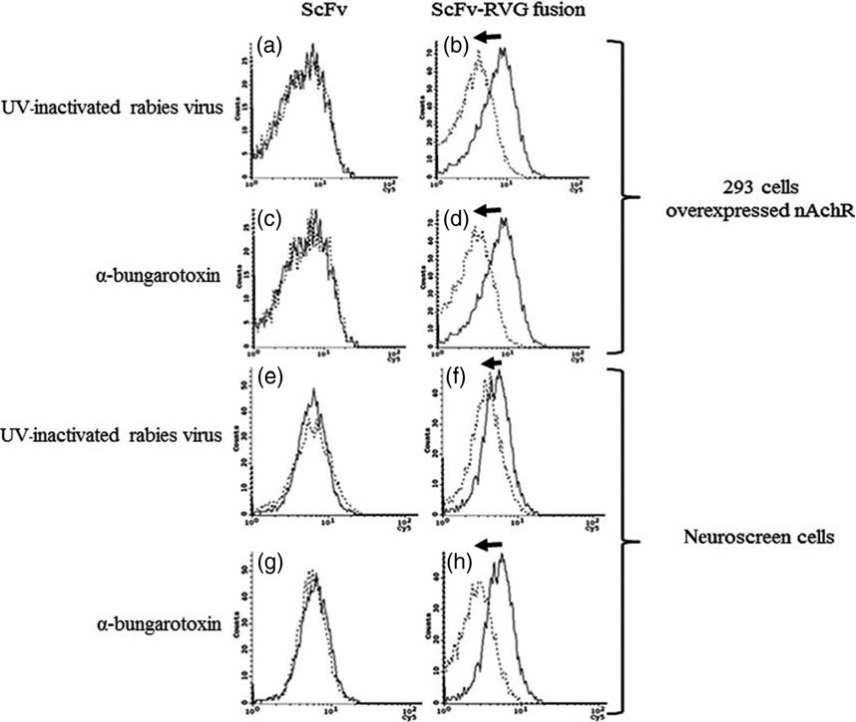
Inhibition of binding penetration of ScFv‐RVG fusion into nAchR overexpressing 293 cells and neuroscreen cells by UV‐inactivated RABV and α‐bungarotoxin. Flow cytometry on nAchR overexpressing 293 cells pretreated with UV‐inactivated RABV (a and b) and α‐bungarotoxin (c and d) before incubation with ScFv (a and c) and ScFv‐RVG fusion protein (b and d). Flow cytometry on neuroscreen cells pretreated with UV‐inactivated RABV (e and f) and α‐bungarotoxin (g and h) before incubation with ScFv (e and g) and ScFv‐RVG fusion protein (f and h). Solid line: no inhibitor, dotted line: pretreated with UV‐inactivated RABV or α‐bungarotoxin. The arrows represent the shift to the left of ScFv‐RVG (dotted line) compared to ScFv (solid line).

UV‐inactivated RABV and α‐bungarotoxin were used as competitive inhibitors for the interaction between the RVG peptide and nAchR. Cells pre‐incubated with each inhibitor were tested for their ability to bind and to internalize ScFv and ScFv‐RVG fusion. There was a low‐level background entry of ScFv into cells. This could not be inhibited by pre‐incubation with either UV‐inactivated RABV or α‐bungarotoxin, indicating that its entry is mediated by a nonspecific mechanism (Figure 5a and c). In contrast, the presence of the UV‐inactivated virus or α‐bungarotoxin inhibited the entry of ScFv‐RVG fusion as evidenced by the shift to the left of the dotted line compared to the absence of the competitor (solid line), shown in Figure 5b and d, respectively. These results confirmed that the entry of ScFv‐RVG fusion protein into cells occurred via a nAchR‐mediated pathway. These experiments were repeated with similar results using a second cell line, neuroscreen cells (Greene and Tischler, 1976), which are neuronal cells that express nAchRs (Figure 5e–h).

### Passage of ScFv and ScFv‐RVG fusion across an *in cellulo* model of the blood–brain barrier

**Figure 6:**
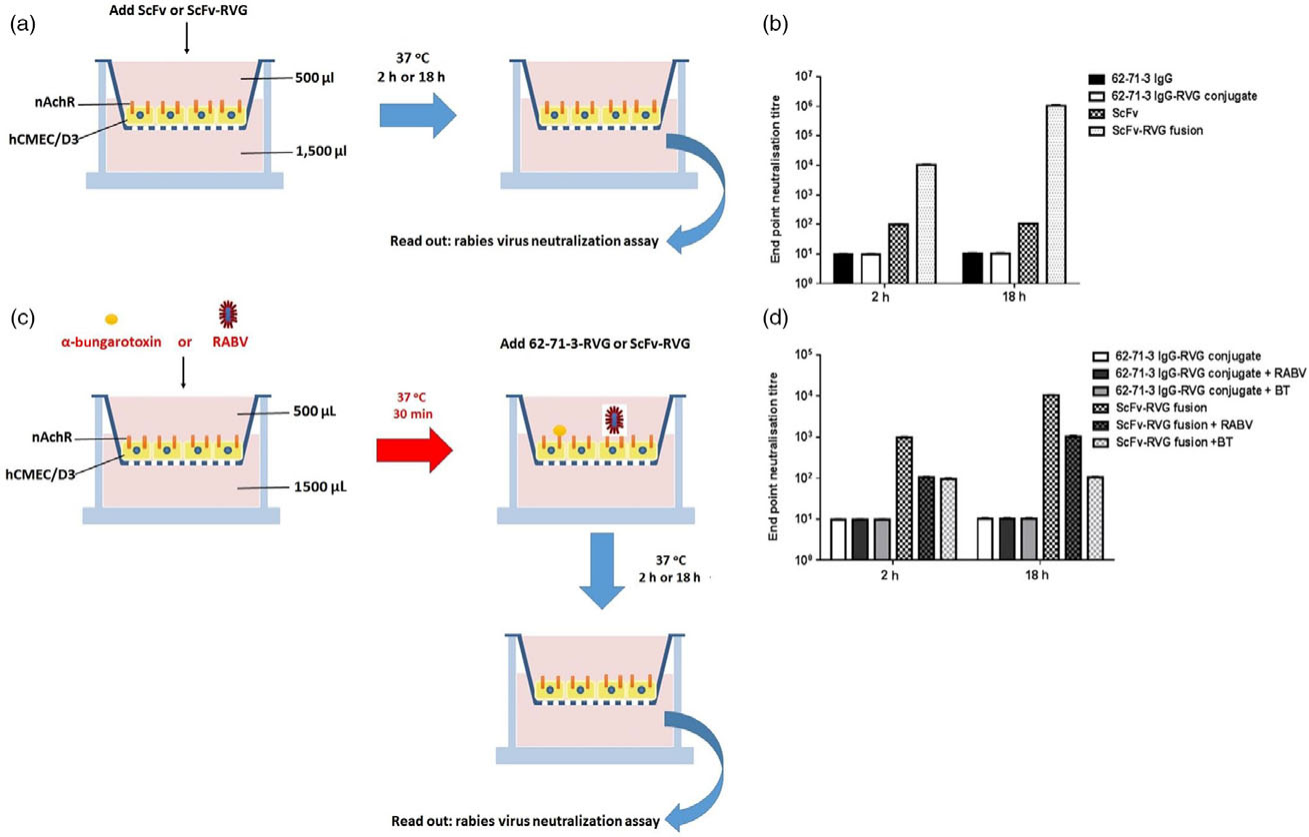
ScFv‐RVG fusion transports across *in cellulo* BBB model. (a) A schematic diagram of the experiment. 10 μg of antibodies was added to the upper chamber of hCMEC/D3 cells in the transwell. Medium (collected after 2 or 18 h after adding the molecules) at the bottom of the well was tested for the presence of RABV‐neutralizing antibodies by a RABV neutralization assay. (b) RABV neutralization titre of 62‐71‐3 IgG, 62‐71‐3 IgG‐RVG conjugate, ScFv and ScFv‐RVG fusion that crossed hCMEC/D3 cells. Each column represents the average values from three independent experiments, and the error bars indicate for the standard deviation (SD). (c) A schematic diagram of the inhibition experiment. hCMEC/D3 cells were pretreated with either UV‐inactivated RABV or α‐bungarotoxin (BT), and then, 10 μg of 62‐71‐3 IgG‐RVG conjugate or ScFv‐RVG fusion was added to the upper chamber. Medium at the bottom of the well was tested for the presence of RABV‐neutralizing antibodies by a RABV neutralization assay after 2 and 18 h. (d) RABV neutralization titre of 62‐71‐3 IgG‐RVG and ScFv‐RVG fusion, which crossed hCMEC/D3 cells after the cells were pretreated with or without RABV or BT. Each column represents the average values from three independent experiments, and the error bars indicate the standard deviation (SD).

The human hCMEC/D3 cell line, which retains morphological and functional characteristics of brain endothelium, is widely used as a human *in cellulo* BBB model (van der Helm *et al*., 2016). The *in cellulo* BBB transport experiment was conducted on the transwell device made with hCMEC/D3 cell monolayer as described in Figure 6 (Eigenmann *et al*., 2013). The barrier integrity of the human brain endothelial cell monolayer was assessed by transport of the small molecule Lucifer yellow and was determined to be 2.11 × 10^−3^ cm/min, attesting to the tightness of the junctions (Figure S1, Siflinger‐Birnboim *et al*., 1987). The expression of nAchR alpha7 on hCMEC/D3 was also confirmed by real‐time PCR (Figure S2). After antibodies were added to the upper chamber, the medium in the lower chamber was tested for RABV‐neutralizing activity after 2 and 18 h (Figure 6a). These time points were chosen to eliminate the caveat of BBB alteration after adding the molecule (i.e. a transport after 2 h only is a very active transport across the endothelial cell barrier). The full‐length 62‐71‐3 mAb did not cross the hCMEC/D3 monolayer, consistent with a previous report for an antibody molecule (Markoutsa *et al*., 2011). 62‐71‐3 IgG‐RVG conjugate did not cross the endothelial cell barrier either (Figure 6b). Some ScFv was found to cross the hCMEC/D3 cells as the 2 h medium sample had neutralizing activity (at dilution 1 : 100), but this did not increase by 18 h (Figure 6b). In contrast, ScFv‐RVG fusion passed through the hCMEC/D3 cells to a much greater extent, and the neutralizing activity of the medium in the bottom well increased in a time‐dependent manner (Figure 6b).

In a second assay, UV‐inactivated RABV and α‐bungarotoxin were used as competitive inhibitors (Figure 6c). Both are natural ligands of α7 nAchR. As before, the 62‐71‐3 IgG‐RVG did not cross the hCMEC/D3 barrier, but the ScFv‐RVG fusion did accumulate in the bottom well in a time‐dependent manner. Pretreating cells with either UV‐inactivated RABV or α‐bungarotoxin reduced the passage of ScFv‐RVG fusion at 2 and 18 h, resulting in at least 10‐fold reduction in neutralizing activity found in the medium in the bottom well (Figure 6d). These inhibitors had no effect on the transport of 62‐71‐3 IgG‐RVG across the barrier (Figure 6d).

## Discussion

Several strategies for the transport across the BBB by drugs or antibodies have been proposed, including association with an antibody recognizing transferrin receptor as a carrier (Friden *et al*., 1991; Pardridge, 2015), targeting to the insulin receptor (Boado *et al*., 2010; Pardridge *et al*., 1985) and formulation with low‐density lipoproteins to target the endothelial LDL‐receptor (Alyautdin *et al*., 1997, 1998; Gulyaev *et al*., 1999). For plant‐manufactured products, cholera toxin B subunit (CTB) was also used successfully to deliver proteins accross the BBB (Kohli *et al*., 2014; Kwon and Daniell, 2016) or to act as a strong mucosal adjuvant (Roy *et al*., 2010; Shahid and Daniell, 2016). Several proteins were used previously to target drugs to the brain, such as the human immunodeficiency virus TAT protein (Schwarze *et al*., 1999) and RVG peptide (Kumar *et al*., 2007; Liu *et al*., 2009). The RVG peptide constitutes part of the mature rabies viral glycoprotein (Kim *et al*., 2013) that can be visualized as trimeric peplomers on the surface of the virion and was previously shown to enable the transvascular delivery of siRNA to the brain (Kumar *et al*., 2007). The region of the viral G protein utilized here, as a linear peptide, has a similar amino acid composition to snake venom α‐bungarotoxin (Lentz, 1991), which was previously shown to bind to nicotinic acetylcholine receptors (nAchR). These receptors are important as they occur in high density at the neuromuscular junction, and are present in the central nervous system and on endothelial cells. Thus, in the case of α‐bungarotoxin, these receptors are also involved in penetration of the toxin into the brain (Bracci *et al*., 1988; Donnelly‐Roberts and Lentz, 1989; McQuarrie *et al*., 1976; Tzartos and Changeux, 1983). Similarly, the full‐length RABV glycoprotein has been shown to interact with nAchR, allowing virus entry into the brain (Burrage *et al*., 1985; Lentz, 1990; Rustici *et al*., 1989).

Size is a key factor governing the ability of a molecule to pass the BBB (Jekic, 1979). 62‐71‐3 ScFv was used in this study because ScFvs are small molecules that retain the antigen specificity of the original immunoglobulin (Bird *et al*., 1988). The neutralization activity of the plant‐produced 62‐71‐3 ScFv had previously been confirmed (Both *et al*., 2013). In this study, 62‐71‐3 ScFv was produced in *N*. *benthamiana* by transient expression at high yields whereas the ScFv‐RVG fusion protein was expressed at significantly lower levels (Figure 2). Similar differences in expression levels between the two molecules were also observed in *Escherichia coli* (data not shown). Moreover, there is a degraded product in the purified protein, which is approximately half the size of the full protein. This degraded product appeared in the immunoblot, confirming the presence of the E tag. This fragment might be either the functional ScFv or the dsRed portion. However, only the band of full‐length protein was used to quantify the amount of molecules used for the next studies for both ScFv and ScFv‐RVG proteins.

Although there are several rabies vaccines and antibodies developed from plants (Hefferon, 2013; Rosales‐Mendoza, 2015; Shahid and Daniell, 2016), here we show for the first time that a fusion protein with the RVG peptide can be produced in plants. Producing the RVG peptide fusion protein in this manner will remove the conjugation step and potentially reduce production costs. Both ScFv and ScFv‐RVG fusion demonstrated equivalent neutralization of live RABV *in cellulo*, indicating that the ability of ScFv to neutralize the virus was not impaired by fusion to the RVG peptide.

To test nAchR binding, HEK293 cells overexpressing nAchR were used (Yamauchi *et al*., 2011). The ScFv‐RVG fusion showed an increase in binding and penetration to cells overexpressing nAchR, compared to ScFv. To confirm that the increase in entry was due to binding to nAchR, both UV‐inactivated RABV and α‐bungarotoxin were used independently as competitive inhibitors. α‐bungarotoxin has a similar structure to RVG and binds to nAchR at the same site as rabies glycoprotein (Donnelly‐Roberts and Lentz, 1989; Lentz, 1991; Lentz *et al*., 1984, 1987, 1988). This investigation demonstrated that entry of ScFv‐RVG fusion into nAchR overexpressing cells decreased when the cells were pretreated with either UV‐inactivated RABV or α‐bungarotoxin, confirming the role of RVG peptide in mediating cell entry via the nAchR.

The BBB possesses specific characteristics that protect the brain from exposure to both endogenous and exogenous toxins. However, this protective barrier also limits the delivery of therapeutic molecules to the brain, a major constraint in developing suitable tools to neutralize RABV that is replicating in the CNS. The gold standard for studying transport across the BBB is to use *in vivo* animal models, but they are expensive, laborious, ethically contentious and often lack predictive data. Therefore, any researcher planning to use animals in their research must first show why there is no alternative to animal experimentation (European Commission, directive 201/63/EU) in order to fulfil the guiding principles underpinning the human use of animals in scientific research (i.e. the three Rs: **R** eplace, **R** educe and **R** efine). Previous study suggested that *in cellulo* models are robust, reproducible, easy to analyse and allow study of human cells and tissues (van der Helm *et al*., 2016) following the 3Rs rules. An *in cellulo* model was, therefore, used here to determine, in a first instance, the potential for the antibodies to cross the human BBB.

The hCMEC/D3 cell line has been developed as a model for the human BBB and has been used to test the permeability of several drugs (Al‐Shehri *et al*., 2015; Ma *et al*., 2014). Here, the results indicated that 62‐71‐3 ScFv was able to pass across the hCMEC/D3 cells whilst the 62‐71‐3 IgG was not. Although the incubation time was increased from 2 to 18 h, the amount of ScFv crossing the cells did not increase. This might be due to the ScFv molecule crossing the cells by passive transport mechanisms and is probably a reflection of the smaller size of this molecule compared to the IgG control. However, when the ScFv was fused with the RVG peptide, the penetration across the cells was significantly increased (Figure 6b) and occurred in a time‐dependent manner indicating active penetration. When competitive inhibitors, UV‐inactivated RABV and α‐bungarotoxin, were used, transport across the *in cellulo* BBB decreased for the ScFv‐RVG fusion protein (Figure 6c) suggesting that the ScFv‐RVG fusion was transported across the *in cellulo* BBB by active transport mechanisms involving binding to nAchR.

Although postexposure prophylaxis in rabies is highly effective when correctly administered, significant challenges remain in treatment of infection, particularly when patient presentation is delayed. Alternative approaches to the treatment of late‐stage rabies infection are still urgently required. The data presented here indicate a potential strategy to deliver potently neutralizing monoclonal antibody fragments across the BBB and into the CNS. Additional *in vivo* animal studies are required to assess pharmacokinetics of ScFv linked to RVG and efficacy of this form of postexposure tool following clinical presentation in an *in vivo* model. This approach may lead to a new mechanism by which postexposure tools can be administered to individuals exhibiting clinical rabies.

## Experimental procedures

### Genetic construct design

The 62‐71‐3 IgG was previously described (Both *et al*., 2013). For the cloning of pEAQ‐ScFv, primers 1, 2, 3, 4, 5, 6, 7 and 8 were used (Figure 1; for the sequences see Table S1). Primer 1 was designed to introduce the attB recombination sites and the *Oryza sativa* signal peptide into the V_H_ domains of mAb 62‐71‐3. Primer 2 was used as a reverse primer for linking the V_H_ and V_L_ domains of mAb 62‐71‐3 with the (Gly_4_Ser)_3_ linker. Primers 3 and 4 were used as forward and reverse primers to amplify V_L_ domains of mAb 62‐71‐3 with NotI site at the 3' end. The V_H_ and V_L_ domains of mAb 62‐71‐3 with the (Gly_4_Ser)_3_ linker were linked using overlap PCR using primers 1 and 4. Primers 5 and 6 were used as forward and reverse primers, respectively, to amplify His tag‐ E tag fusion gene containing NotI and BamHI sites. Primer 7 was used as a forward primer to amplify dsRed gene containing BamHI site. Primer 8 was used as a reverse primer to amplify dsRed and also contained attB recombination sequence to the 3' end of dsRed gene. dsRed gene was included to monitor ScFv/ScFv‐RVG expression in cells by immunofluorescence. The V_H_ and V_L_ domains of mAb 62‐71‐3 with the (Gly_4_Ser)_3_ linker were digested with NotI restriction enzyme. The fusion His tag ‐ E tag portion was digested with NotI and BamHI restriction enzymes. The dsRed gene was digested with BamHI restriction enzyme. These three pieces were ligated, purified using the QIAquick PCR purification kit (Qiagen) and recombined into the Gateway entry vector pDONR/Zeo (all materials for Gateway recombination including enzymes, entry vector pDONR/Zeo, competent *E*. *coli* cells and zeocin, were obtained from Invitrogen). The *E*. *coli* cloning strain DH5α was heat‐shocked with the plasmids and streaked on plates containing LB plus 50 μg/mL zeocin. Individual colonies were used for inoculating 5 mL LB medium containing 50 μg/mL zeocin and were shaken overnight (250 rpm, 37 °C). The plasmids were purified from a saturated overnight culture with the QIAprep Spin Miniprep Kit (Qiagen) and used for recombination with the Gateway destination vector pEAQ‐HT‐DEST3 (Sainsbury *et al*., 2009). For the cloning of pEAQ‐ScFv‐RVG (Figure 1), the V_H_ and V_L_ domains of mAb 62‐71‐3 with the (Gly_4_Ser)_3_ linker and the His tag – E tag portion were cloned using the same method as pEAQ‐ScFv. Primers 9 and 10 were used as forward and reverse primers, respectively, to amplify RVG peptide with BamHI site at the 5' end. Primers 11 and 8 were used as forward and reverse primers, respectively, to amplify dsRed gene. The RVG peptide and dsRed genes were linked by overlap PCR using primers 8 and 9. After the three pieces were ligated, the Gateway recombination was performed using the same method as previously described for pEAQ‐ScFv.

### Plant inoculation and protein expression

*Agrobacterium tumefaciens* LBA4404 was transformed with the pEAQ‐ScFv and the pEAQ‐ScFv‐RVG fusion vectors by electroporation. Recombinant bacterial strains were used to infiltrate leaves of *N*. *benthamiana* plants under vacuum. Leaves were harvested on days 4, 5, 6 or 7 postinfiltration for expression time‐course experiments. For other experiments, the leaves were harvested on day 6 postinfiltration. Soluble proteins were extracted in 0.1m Tris‐HCl pH 7.5 + 0.2% Triton X, using a blender before centrifugation at 18 000 ***g*** for 10 min. The supernatant was retained for analysis.

### SDS‐PAGE and western blot

Plant extracts were denatured by boiling in NuPAGE^®^ LDS Sample Buffer and separated on 4%–12% polyacrylamide gels (Life Technologies, Warrington, UK). Proteins were either visualized by Coomassie blue staining or transferred to a nitrocellulose membrane (Amersham Hybond‐ECL; Amersham Biosciences, Little Chalfont, UK). The membrane was blocked with 5% nonfat dried milk, 0.1% Tween 20 in PBS. The membrane was probed with horseradish peroxidase (HRP)‐conjugated mouse anti‐His tag antiserum (Sigma) or HRP‐conjugated mouse anti‐E tag antiserum (Abcam, Cambridge, UK) diluted at 1 : 5000 in 1% nonfat dried milk in PBST. The membranes were developed by chemi‐luminescence using ECL plus detection reagent (GE Healthcare, Buckinghamshire, UK).

### Protein purification

Plant extract was filtered through Miracloth (EMD Millipore, Massachusetts, USA), centrifuged at 20 000 *g* for 15 min and passed through a 0.2‐μm filter (Merck Millipore, Germany). Purification was by Ni‐affinity chromatography with chelating SepharoseTM (GE healthcare) charged with NiSO4.6H2O. The antibody molecules were extensively purified from the crude extract, but this affinity purification method does not reach a purification at homogeneity.

### Cells and viruses

BSR cells (a clone of baby hamster kidney (BHK) cells) were grown in Dulbecco's modified Eagle's medium (DMEM)‐Glutamax I (Life Technologies) supplemented with 10% foetal calf serum and penicillin/streptomycin. Neuroscreen cells (a subclone of PC12 cells, Cellomics, USA, which express α7‐nicotinic acetylcholine receptor) were grown in RPMI medium (Sigma, Welwyn Garden City, UK) supplemented with 10% horse serum, 5% foetal calf serum and penicillin/steptomycin. Human Embryonic Kidney 293 (HEK) cells overexpressing human α7‐nicotinic acetylcholine receptor (nAchR) (Yamauchi *et al*., 2011) were grown in DMEM supplemented with 10% foetal calf serum and Pen/Strep. The nAchR expression in this cell line was monitored by flow cytometry (data not shown). The hCMEC/D3 cells were grown in EndoGro™ medium (Millipore, Molsheim, France) according to the manufacturer's instruction. The nonpathogenic RABV laboratory strain ERA was propagated as previously described (Thoulouze *et al*., 1997).

### *In cellulo* RABV neutralization assay

Neutralization of the ERA strain was performed on BSR cells using the rapid fluorescent focus inhibition test (Louie *et al*., 1975). The negative control consisted of medium without antibody. Dilutions of the test antibodies were incubated with RABV(<20 PFU) for 1 h at 37 °C before incubating with BSR cells at 37 °C with 5% CO2. After 48 h, the supernatant was removed and the cells were fixed with 80% acetone at 4 °C for 30 min. The cells were washed and incubated with 1 : 50 FITC‐conjugated mouse anti‐RABV nucleocapsid antiserum (Bio‐Rad, Marnes‐la‐Coquette, France) at 37 °C for 30 min. After washing, RABV foci were counted using a fluorescent microscope. Assays were performed in triplicate.

### nAchR binding and competition assay

HEK 293 cells expressing human α7‐nicotinic acetylcholine receptor (nAchR) (Yamauchi *et al*., 2011) or Neuroscreen cells (entry assay) were seeded on six‐well plates. After 24 h, cells were placed on ice and treated with ScFv preparations for 5 or 30 min, for the binding and entry assays, respectively. Of note, over a 5‐min incubation on ice, it is expected that only a few single‐chain antibody molecules are able to penetrate into the cell (Lim *et al*., 2013). After washing, the cells were harvested and incubated in cell fixation solution (BD Biosciences) for 15 min. For the binding assay, samples were washed with 1% inactivated foetal calf serum and 0.1% NaN3 in PBS, pH 7.4, whilst for the binding‐penetration assay, samples were washed with 1% inactivated foetal calf serum, 0.1% NaN_3_ and 0.1% saponin in PBS, pH 7.4, before incubation with 1 : 1000 mouse anti‐E tag antiserum at 4 °C, overnight. The cells were washed and incubated with goat anti‐mouse IgG antiserum conjugated with cy5 (Jackson laboratory, West Grove, Pennsylvania, USA) at 37 °C for 1 h. The absence of saponin in the binding assay allowed us to detect the ScFv cytoplasmic membrane bound molecule as the secondary antibody is not able to penetrate inside the cell. Again, the cells were washed, resuspended in staining buffer and analysed with FACS CellQuest software (BD, US). Alternatively for a competition assay, cells were pretreated with either 2x10^7^ PFU of UV‐inactivated RABV'Challenge Virus Strain' (CVS) (Megret *et al*., 2005) (i.e. still able to bind to RABV receptors but not replicative) or 16 μm α‐bungarotoxin (Tocris Bioscience, Bristol, UK) for 30 min on ice, before the ScFv or ScFv‐RVG fusion was added.

### *In cellulo* BBB transwell assay

The hCMEC/D3 cell line was prepared as described (Eigenmann *et al*., 2013) and seeded on the apical side of a Cultrex^®^ Rat Collagen I (150 μg/mL‐R&D Systems, Minneapolis, Minnesota, USA) coated 0.9 cm^2^ polyethylene terephthalate filter insert with 3.0 μm porosity (BD Falcon, Loughborough, UK). 10 μg of each antibody preparation was added to the top chamber. The cells were incubated at 37 °C with 5% CO2, and the medium was sampled after 2 h and 18 h from the bottom chamber for neutralizing antibody detection. For inhibition of *in cellulo* BBB penetration, UV‐inactivated RABV or α‐bungarotoxin was added to the top chamber for 30 min before 62‐71‐3 IgG‐RVG conjugate and ScFv‐RVG fusion protein were added. The medium in the bottom chamber was sampled as before at 2 h and 18 h.

### Determination of the restrictive paracellular permeability with Lucifer Yellow

The restrictive paracellular permeability of hCMEC/D3 cells was assessed by their low permeability to the nonpermeant fluorescent marker Lucifer Yellow (LY) (Sigma‐Aldrich, L0259). Briefly, after 5 days of culture on filters, hCMEC/D3 monolayers were transferred to 12‐well plates containing 1.5 mL of transport medium (HBSS CaMg (Gibco, 14025‐100) supplemented by 10 mm of hepes (Life technologies, 15630‐080) and 1 mm of sodium pyruvate (Life technologies, 11360)) per well (abluminal compartment). 0.5 mL transport medium containing 50 μm of LY was then added to the luminal compartment. Incubations were performed at 37 °C, 5% CO_2_ and 95% humidity. After 15, 25 and 45 min, the inserts were transferred into new wells, beforehand filled with 1.5 mL of transport medium. After 45 min, aliquots were taken for each time point, from both compartments and the concentration of LY determined using a fluorescence spectrophotometer (Tecan Infinite F500).

The endothelial permeability coefficient (P_e_) of LY was calculated in centimetres/min (cm/min), as described previously (Siflinger‐Birnboim *et al*., 1987). To obtain a concentration‐independent transport parameter, the clearance principle was used. Briefly, the average volume cleared was plotted versus time, and the slope was estimated by linear regression. Both insert permeability (PS_f_, for insert only coated with collagen) and insert plus endothelial cell permeability (PS_t_, for insert with collagen and cells) were taken into consideration, according to the following formula: 1/PSe=1/PSt−1/PSf.

The permeability value for the endothelial monolayer was then divided by the surface area of the porous membrane of the insert (Corning, 3460) to obtain the endothelial permeability coefficient (Pe) of the molecule (in cm/min).

### Quantitative polymerase chain reaction

The procedure undertaken has been described in Chopy *et al*. (2011). Basically, cDNA synthesis was performed with 1 μg total RNA using SuperScript II reverse transcriptase (Life Technologies, France). Quantitative real‐time RT‐PCR (qRT‐PCR) was performed in triplicate using an ABI Prism 7500 fast sequence detector system (primers 18S: F: CTT AGA GGG ACA AGT GGC G, R: ACG CTG AGC CAG TCA GTG TA; α7 AchR QT00074732, Qiagen, France) with GoTaq PCR master mix (Promega, Charbonniéres‐les‐Bains, France). After normalization to 18S rRNA, the relative abundance of mRNA was obtained by calculation of the difference in threshold cycles of the test and control samples (mock value set to 1), commonly known as the ΔΔC_T_ method.

## Supporting information


**Figure S1** Integrity of the BBB device was assessed by measuring the permeability to Lucifer Yellow. Data are representative of three independent filters (mean ± SD).
**Figure S2** Bar graph illustrating real‐time PCR data demonstrating the expression of alpha7 subunit AchR by hCMEC/D3 (human endothelial cell line from brain microvessels) and SH‐SY5Y (human neuroblastoma) cells.
**Table S1** PCR primers for cloning pEAQ‐ScFv and pEAQ‐ScFv‐RVG.
