# Supplementary material for: Enhanced transport of plant‐produced rabies single‐chain antibody‐RVG peptide fusion protein across an in cellulo blood–brain barrier device
Source: Plant Biotechnol J. 2017 Apr 19;15(10):1331–9. doi: 10.1111/pbi.12719 (PMC5595719; doi:10.1111/pbi.12719)
Supplement: Supplementary file 1 — Figure S1 Integrity of the BBB device was assessed by measuring the permeability to Lucifer Yellow. Data are representative of three independent filters (mean ± SD). Figure S2 Bar graph illustrating real‐time PCR data demonstrating the expression of alpha7 subunit AchR by hCMEC/D3 (human endothelial cell line from brain microvessels) and SH‐SY5Y (human neuroblastoma) cells. Table S1 PCR primers for cloning pEAQ‐ScFv and pEAQ‐ScFv‐RVG. [file PBI-15-1331-s001.docx]

**Supplementary figure S1.** Integrity of the BBB device was assessed by measuring the permeability to Lucifer Yellow.

Data are representative of three independent filters (mean +/- SD).

**Supplementary figure S2.** Bar graph illustrating real-time PCR data demonstrating the expression of alpha7 subunit AchR by hCMEC/D3 (human endothelial cell line from brain microvessels) and SH-SY5Y (human neuroblastoma) cells.


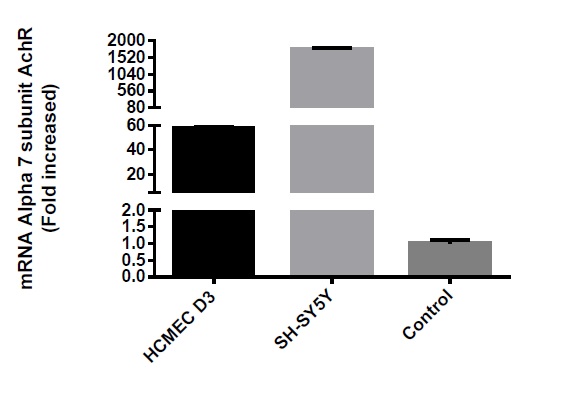


**Supplementary** **table 1.** PCR primers for cloning pEAQ-ScFv and pEAQ-ScFv-RVG

| No. | Primer name | 5’ 🡪 3’ Sequence |
| --- | --- | --- |
| 1 | attB-Os-F | GGGGACAAGTTTGTACAAAAAAGCAGGCTCAACCATGGGGAAGCAAATGGCCGCCCTGTGTGGCTTTCTC |
| 2 | VH-Linker-R | CCGCCACCTGAACCGCCACCACCAGATCCACCACCACCTGAAGAAACAGTGAGAGTAG |
| 3 | Linker-VL-F | TGGATCTGGTGGTGGCGGTTCAGGTGGCGGAGGTTCTGATGTTCAAATGACTCAG |
| 4 | NotI-VL-R | GCGGCCGCTCTCTTAATCTCAAGCTTAGTGC |
| 5 | NotI-6His-F | GCGGCCGCACATCATCACCACCATCAC |
| 6 | BamHI-Etag-R | GGATCCGCTCTACCACCCTTATACTC |
| 7 | BamHI-dsRed-F | GGATCCTGCATTTCTATATAAGGTTGTGGATGGAATGGGCTCCTCCAAGAACG |
| 8 | attB-dsRed-R | GGGGACCACTTTGTACAAGAAAGCTGGGTTCAAAGGAACAAGTGATGC |
| 9 | BamHI-29RVG-F | GGATCCTGCATTTCTATATAAGGTTGTGGATGGATACACTATCTGGATGCCAG |
| 10 | 29RVG-dsRED-R | CGTTCTTGGAGGAGCCCATTCCATTAGAAGCCCTCTTG |
| 11 | 29RVG-dsRED-F | CAAGAGGGCTTCTAATGGAATGGGCTCCTCCAAGAACG |
